# A stochastic model of active zone material mediated synaptic vesicle docking and priming at resting active zones

**DOI:** 10.1038/s41598-017-00360-z

**Published:** 2017-03-21

**Authors:** Jae Hoon Jung, Sebatian Doniach

**Affiliations:** 1 Department of Physics, Stanford University School of Humanities and Sciences, Stanford, CA 94305 USA; 2 Department of Biology, Texas A&M University, College Station, TX 77843 USA

## Abstract

Synaptic vesicles (SVs) fuse with the presynaptic membrane (PM) at specialized regions called active zones for synaptic transmission. SVs are associated with dense aggregates of macromolecules called active zone material (AZM) that has been thought to be involved in SV release. However, its role has recently begun to be elucidated. Several morphological studies proposed distinctively different AZM mediated SV docking and priming models: sequential and concurrent SV docking/priming. To explore ways to reconcile the contradictory models we develop a stochastic AZM mediated SV docking and priming model. We assume that the position of each connection site of the AZM macromolecules on their SV, directly linking the SV with the PM, varies by random shortening and lengthening of the macromolecules at resting active zones. We also perform computer simulations of SVs near the PM at resting active zones, and the results show that the distribution of the AZM connection sites can significantly affect the SV’s docking efficiency and distribution of its contact area with the PM, thus priming and that the area correlates with the shape of the SVs providing a way to account for seemingly irreconcilable observations reported about the spatial relationship of SVs with the PM at active zones.

## Introduction

Synaptic vesicles (SVs) dock and fuse at specialized regions called active zones on the presynaptic plasma membrane (PM) of axon terminals for synaptic transmission^1–3^. The active zone contains dense aggregates of cytoplasmic macromolecules called active zone material (AZM; also called membrane thickenings, presynaptic dense projections or cytomatrix)^4–8^. When an action potential arrives at the active zone, calcium channels in the PM open, and the influx of calcium ions through the channels from the extracellular space triggers a certain number of SVs, which have undergone priming, a process of rendering vesicles fusion-ready^9, 10^, to fuse with the PM. The fused SVs release chemical signals called neurotransmitters, which are stored in the SVs to elicit a postsynaptic response for synaptic transmission^1, 11^.

**Figure 1:**
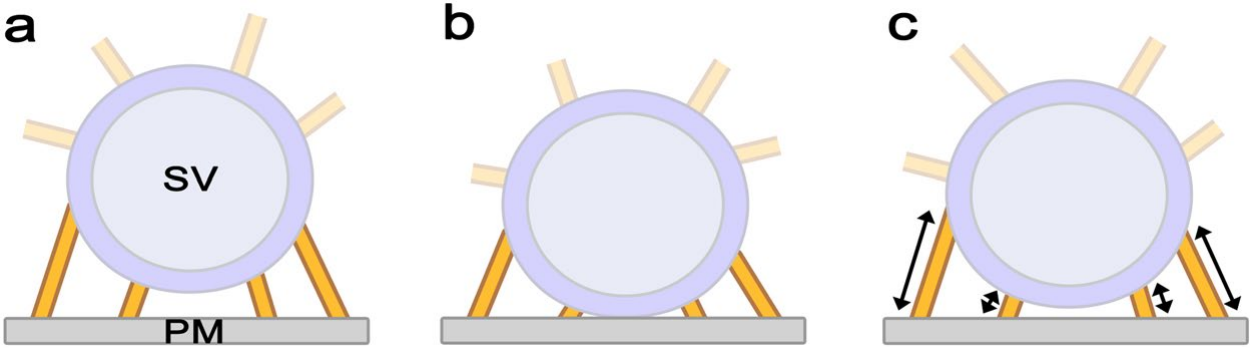
Schematic diagrams of synaptic vesicles (SVs) in the vicinity of the presynaptic membrane (PM) at active zones and a stochastic active zone material (AZM) mediated SV docking and priming model. Two schematic diagrams on the left depict SVs in the vicinity of the PM typically found at active zones. Multiple AZM macromolecules (yellow) are associated with the SVs, and several AZM macromolecules directly link the SVs to the PM while other AZM macromolecules link the SVs to the PM indirectly. It should be noted that non-AZM macromolecules are also found linking the SVs to other SVs^15, 20^, which are not shown here. (**a**) An undocked SV near the PM associated with multiple AZM macromolecules directly linking the SV to the PM (golden) and other AZM macromolecules (semitransparent golden). (**b**) A docked SV with the PM associated with multiple AZM macromolecules (golden) directly linking the SV to the PM and other AZM macromolecules (semitransparent golden). (**c**) A stochastic AZM-mediated SV docking and priming model based on the hypothesis that the AZM macromolecules directly linking SVs to the PM randomly shorten and lengthen. The model proposes that the SV docking and priming are regulated by the forces generated via the structural changes of the AZM macromolecules that contain, at least in part, proteins crucial for SV fusion with the PM.

The AZM has long been thought to be involved in SV docking and priming for fusion due to its association with SVs at the active zone^6, 12^. After the development of electron tomography that can provide detailed structural information in 3-dimension (3D) at several nanometer resolution, electron tomography directly visualized SVs associated with multiple AZM macromolecules and the PM at active zones^4, 12–19^ and showed that undocked SVs at the active zone closely located to the PM are also connected to multiple AZM macromolecules^4, 13, 17^ as depicted in Fig. 1. The distance from the undocked SVs to the PM was found to be negatively correlated with the number of the AZM connections indicating that the association of the AZM with an SV is involved in SV docking^17, 18^. However, an electron tomography study on synapse preparations from rat brain showed that almost all of the observed SVs at active zones have notable gaps between the SVs and the PM while they are associated with several short AZM macromolecules (or tethers) suggesting that the short AZM macromolecules play an important role on SV priming and that the direct contact between the vesicle membrane and the PM only occurs during the process of fusion between the membranes^20^.

In contrast, a recent electron tomography study on resting frog’s neuromuscular junctions showed that most of SVs at active zones are in contact with the PM and that the contact area of the docked SVs with the PM has a broad variation more than 10-fold and correlates with the length and position of several classes of the AZM, which were found to show normal distributions indicative of their random variation^21–23^, proposing that the contact area of a docked SV is regulated by random shortening and lengthening of the classes of the AZM and that the extent of the contact area is a morphological indicator of priming for docked SVs; intriguingly, only a small portion of SVs near the PM were found to be undocked (~2%) whereas the vesicles are connected to all the classes of the AZM similar to docked SVs^4^. On the other hand, numerous undocked SVs near the PM at active zones in addition to docked SVs have been observed in synaptic nerve terminals of rat and mouse brains^13, 18, 24^. Thus, it remains unclear whether the SV docking and priming are sequential or concurrent; the findings may indicate that morphological characteristics of SV docking and priming depend on the kind of synapses and/or the type of species despite the widely observed association of multiple AZM macromolecules with SVs in vicinity of the PM at active zones.

We note that the hypothesis of random shortening and lengthening of the AZM for priming of docked SVs^4^ can be also applicable to undocked SVs near the PM at active zones. Thus, in this study we extend the hypothesis to make it applicable to undocked SVs in the vicinity of the PM as well as docked SVs at the active zone by constructing a stochastic AZM mediated SV docking and priming model (see Fig. 1c). We also explore the model by simulating and analyzing the spatial relationship of SVs with the PM that are adjacent to or docked with the PM and comparing the results with the recent findings from electron tomography studies on frog’s neuromuscular junctions and synaptic nerve terminals of rat and mouse brains^4, 18, 20, 24^. We discovered that the model can provide a way to reconcile the seemingly contradictory observations about the spatial relationship of SVs with the PM at active zones in various synapses. Here we employ a Monte Carlo Markov Chain simulation technique to investigate the movement of SVs contributing to docking and priming at the active zone with sub-nanometer resolution, which cannot be detected by current microscopy techniques^4, 25–27^. The simulation results show that the random variation in the proximities of several AZM macromolecular structures on each SV to the PM can account for the presence of undocked SVs near the PM and also the broad variation in the contact area of docked SVs on the PM at active zones^4, 18, 24^. Moreover, the results suggest that the distribution of the connection sites of the randomly shortening and lengthening AZM macromolecules is an important factor for the regulation of the SV’s docking efficiency and the distribution of the SV’s contact area with the PM. The stochastic AZM-mediated SV docking and priming model proposed here provides a simple mechanism for the role of the AZM on SV docking and priming at active zones, and the model suggests that the direct mechanical coupling of the AZM with the spatial relationship between SVs and the PM brings about variation in the spatial relationship, which may play an important role on the regulation of SV fusion with the PM at the active zone for synaptic transmission.

## Methods

### A stochastic AZM-medicated vesicle docking and priming model

**Figure 2:**
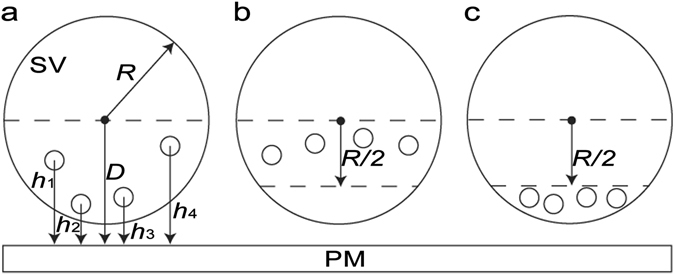
Schematic diagrams of three SVs having different arrangements of multiple connection sites of AZM macromolecules on the SVs and parameters that quantify the spatial relationships of the SVs and the connection sites with the PM. Schematic diagrams show three SVs having different arrangements of multiple AZM macromolecules (open circle) on their hemispherical surfaces facing the PM. Each SV is idealized to be a sphere with a radius, *R*, and the height of each AZM connection site is the distance from the AZM connection site to the PM where *h*_*i*_ is the height of the *i*-th AZM connection site where *i* = 1, 2, …, *n* (the total number of the AZM connection sites). The proximity of each SV’s center to the PM (*D*) is used to determine the spatial relationship between the SV and the PM. Thus, the proximity of each SV is greater than *R* when the SV is undocked from the PM, and the proximity is less than *R* when the SV is docked with the PM. For docked SVs, *D* is used to obtain the contact area of the SVs with the PM. (**a**) Multiple AZM connection sites are randomly distributed on the entire hemisphere of an SV, which is the region below the dotted line on the SV. (**b**) The AZM connection sites are distributed randomly on the upper half hemisphere of an SV, which is the region between the two dotted lines on the SV. (**c**) The AZM connection sites are distributed randomly on the lower half of the hemisphere of a SV, which is the region below the dotted line in the middle of the SV’s hemisphere.

As shown in Fig. 1, our model is based on a recently proposed hypothesis that multiple AZM macromolecules near the contact site of a docked SV randomly shorten and lengthen independently regulating the extent of the contact area that represents the degree of priming^4^. It assumes that multiple AZM macromolecules near not only the contact site of an SV but also the docking site are involved in docking and priming by their random independent shortening and lengthening as shown in Fig. 1c. Realistically, the AZM macromolecules away from the docking site or the contact site of a vesicle cannot contribute as effectively as the macromolecules near the docking site or the contact site through their force-generating shortening. Thus, our model considers only the AZM macromolecules contacting the hemisphere of a docking or docked SV facing the PM, and as suggested by several structural studies^4, 15, 17^, we assume that the connection sites of the AZM macromolecules on the vesicle membrane are relatively stable such that the distance between the SV and the PM is directly regulated by the average distance from the connection sites to the PM (see Fig. 2). Accordingly, our model consists of an SV, the PM, and multiple AZM macromolecules linking the vesicle to the PM, each of which can shorten and lengthen independently. The distribution of the AZM connection sites on a SV might be random as in Fig. 2a, but the distribution can be limited to a specific region as in Fig. 2b,c. Thus, we explore the effect of such different distribution on the spatial relationship of an SV with the PM.

The presence of calcium channels near a SV is critical for synaptic transmission. The number of calcium channels, their position with respect to the SV, and their open probability can greatly contribute to fusion of the SV with the PM^28–30^; however, we ignored the calcium channels because our study is focused on the dynamics of SVs at resting terminals prior to the influx of calcium ions through the channels in proximity to the SV.

Because the AZM is the structural organization that links each SV at the active zone to the PM, the AZM has been widely thought to contain key proteins such as SNARE proteins and their regulatory proteins for SV fusion with the PM^4, 6, 8, 12, 15, 17^; it was recently reported that docked SVs at active zones of frog’ neuromuscular junctions has multiple AZM macromolecules directly linking the vesicles to the PM and that the average distances from connection sites of the AZM macromolecules on the SVs to the PM follow a normal distribution^4^. Accordingly, we assume that the probability distribution of the distance from the connection site of each AZM macromolecule on the vesicle membrane to the PM or the height of the connection site follows a normal distribution; simply, we use a normal distribution for each height (*h*_*i*_) having $\frac{{h}_{i0}}{2}$ as the mean height and $\frac{{h}_{i0}}{6}$ as the standard deviation where *h*_*i0*_ is the initial height of the connection site of the i-th AZM macromolecule:

1 $$N({h}_{i}|\,{\bar{h}}_{i},{\sigma }_{i})=\frac{1}{\sqrt{2\pi {{\sigma }_{i}}^{2}}}{e}^{-\frac{{({h}_{i}-{\bar{h}}_{i})}^{2}}{2{{\sigma }_{i}}^{2}}}$$

where ${\bar{h}}_{i}$, the mean height is $\frac{{h}_{i0}}{2}$ and *σ*_*i*_, the standard deviation is $\frac{{h}_{i0}}{6}$. Here, $\frac{{h}_{i0}}{6}$ is chosen as the standard deviation to limit the distribution of the height largely within the range [0, *h*_*i*0_]. Accordingly, most of the distribution of each height (99.7%) is located in [0, *h*_*i*0_]. The height is expected to constantly change due to the random but gradual shortening and lengthening of the AZM macromolecule. In order to account for its transition, the new height that the current height can take is assumed to have a normal distribution, $N({h}_{ic},{\sigma }_{ic})\,\,$where *h*_*ic*_ is the current height and *σ*_*ic*_ is set to be 0.10*R* where *R* is the radius of a vesicle. Then the transition probability is calculated in the following way using Markov Chain Monte Carlo simulations based on Metropolis-Hastings algorithm^31–33^. Accordingly,

2 $${p}_{t}=\,{\rm{\min }}(1,\frac{{f}_{h}}{{f}_{c}})$$

where *p*_*t*_ is the transition probability, *f*_*h*_ the probability density having *h* as the new height, and *f*_*c*_ the probability density having the current height. The minimum value between 1 and $\frac{{f}_{h}}{{f}_{c}}$ is selected. Thus, if *f*_*h*_/*f*_*c*_ is greater than or equal to 1, the current height changes to the new height. Otherwise, the chance of the transition of the current height to the new height is *f*_*h*_/*f*_*c*_, which is less than 1. Accordingly, a number from a uniform distribution ranging from 0 to 1 is randomly chosen. If the number is less or equal to *f*_*h*_/*f*_*c*_, the current height is replaced by the new height. Otherwise, the height doesn’t change.

All calculations were performed using the Markov Chain Monte Carlo simulations of the model. In our simulations the shape of each SV is idealized to be a sphere with its radius (*R*) set to be 1.0 for simplicity although vesicles are not perfectly spherical in general^4^.

The modeling program was written in IDL (Exelis, Boulder, CO) and executed on computers running under Windows operating systems. We used 80000 iterations for each simulation to ensure that the statistical errors were negligible.

We study three versions of the AZM-medicated SV docking and priming model; a model with the random distribution of multiple AZM macromolecules on the hemisphere of an SV, a model with the random distribution of multiple AZM macromolecules on the upper half hemisphere of an SV, and a model with the random distribution of multiple AZM macromolecules on the lower half hemisphere of an SV.

### Random distribution of multiple AZM macromolecules on the entire hemisphere of a synaptic vesicle facing the presynaptic membrane

We implement the random distribution of multiple AZM macromolecules on the hemisphere of an SV by assuming that the multiple AZM macromolecules involved in docking and priming by their shortening and lengthening are randomly distributed on the hemisphere of an SV. Then, the vesicle is initially located close to the PM and associated with the multiple AZM macromolecules.

To simulate a set of the AZM connection sites on the hemisphere of an undocked SV, points are parameterized by $(\theta ,\,\phi ,\,h)$, where *θ* is the latitude, *φ* the longitude, and *h* the height of each connection site from the PM. To sample the hemispherical surface at points randomly distributed on the hemispherical surface, we first selected the height for each AZM connection site randomly from the uniform distribution ranging from *D*-*R* to *D* where *R* is the radius of an SV and *D* is the distance from the SV’s center to the PM. Then, *θ* is computed given by

3 $$\theta ={\sin }^{-1}(\sqrt{1-\frac{{(D-h)}^{2}}{{R}^{2}}})$$

**Figure 3:**
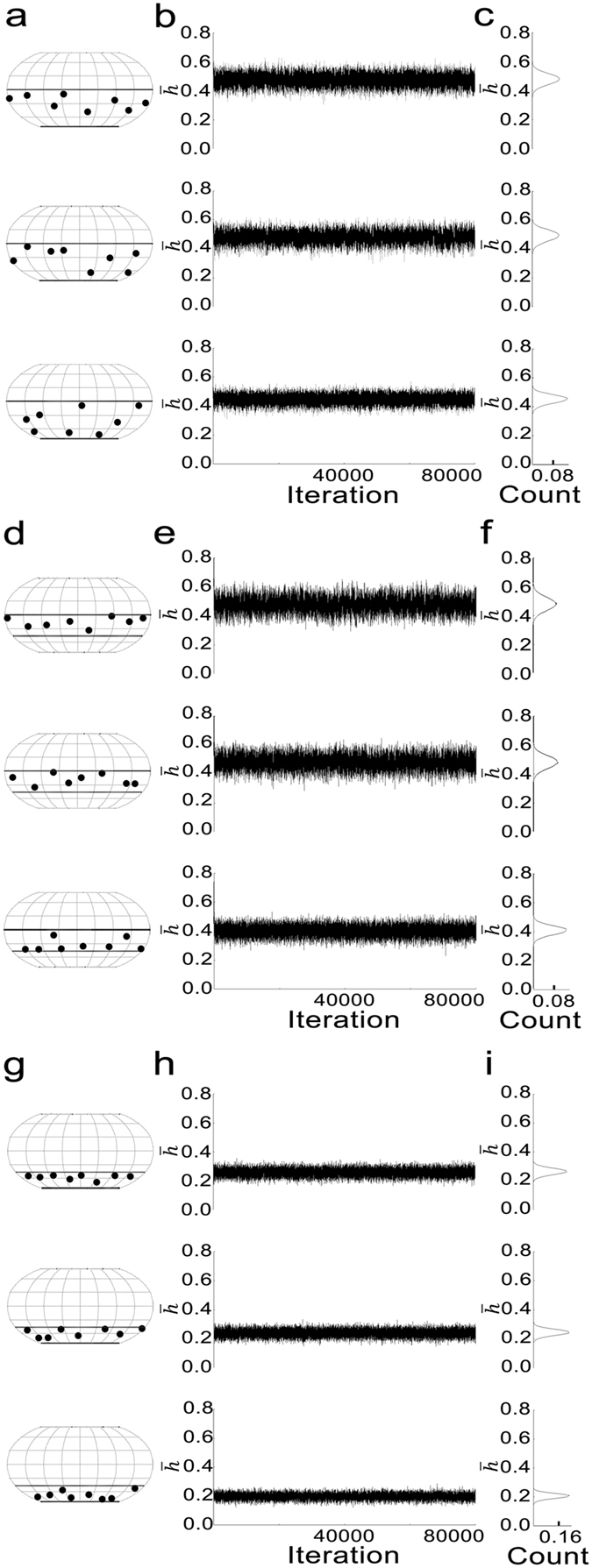
Example distributions of the AZM connection sites on the hemispherical surfaces of nine different SVs, variations in their average heights from the PM, and histograms of the average heights from Markov Chain Monte Carlo simulations. (**a,d,g**) Three different sets of eight AZM connection sites randomly distributed on the surface of an SV’s hemisphere, upper half hemisphere, and lower half hemisphere, respectively plotted on Robinson maps to visualize their locations on their entire vesicle surface. The hemispheres of the vesicles facing the PM lie below the equator drawn in bold dotted lines. (**b,e,h**) Fluctuations in the average height ($\bar{h}$) of each set of the AZM connection sites generated over 80000 iterations using Markov Chain Monte Carlo simulations. (**c,f,i**) Histograms of the average heights for the three different sets of the AZM connection sites from the iterations.

Next, *φ* is selected randomly from the uniform distribution ranging from 0° to 360° in order to fix the connection site on the vesicle. Then each AZM connection site is simulated to randomly fluctuate following a Gaussian distribution with transition probabilities described previously. The process is iterated 80000 times, and we obtained its distribution (see Fig. 3).

Initially, the connection sites of all the AZM macromolecules were randomly distributed on the surface of the SV’s hemisphere facing the PM (see Fig. 3). For each iteration, the best fit of the SV’s center along the z-axis was computed by minimizing the deviation of the distance from the center to each connection site from 1.0, and the proximity of the center of the best fit SV to the PM (*D*) was recorded. Then, the height of each connection site on the best fit sphere from the PM was recorded in each of the iterations, and the dimensionless average height of all the connection sites was calculated using a formula:

4 $$\bar{h}=\frac{{\sum }_{i=1}^{n}{h}_{i}}{nR}$$

where *h*_*i*_ is a height of each connection site, $\bar{h}$ the average height of the AZM connection sites, and *n* the total number of AZM connection sites of an SV. The shape of an SV changes after docking, and the shape of the docked SV on the PM can be determined theoretically by minimizing continuous curves of the membranes, but it can vary depending on specific models and their required input parameters. Thus, for simplicity, the shape of an SV is assumed to remain the same after docking. Then the contact area is obtained by calculating the intersected area of the best fit sphere by the PM and dividing it by the total surface area of the SV. Here we use the dimensionless contact area (*A*), which is given by

5 $$A=\pi ({R}^{2}-{D}^{2})/(4\pi {R}^{2})$$

where *D* < *R*. Accordingly, for each SV, the proximity of its center, the average height of all the AZM macromolecules, and the contact area of the SV were recorded at each of the iterations. Here we used 500 SVs, and their total distributions of each average height, proximity, and the contact area were obtained for comparison analysis.

### Random distribution of multiple AZM macromolecules on the upper half hemisphere of an SV facing the PM

We implement the non-random distribution of multiple AZM macromolecules on the hemisphere of an SV by assuming that the multiple AZM macromolecules are randomly distributed but limited to the upper half hemisphere of a vesicle (see Fig. 2). Then simulations were carried out as described above.

### Random distribution of multiple AZM macromolecules on the lower half hemisphere of an SV facing the PM

We implement the non-random distribution of multiple AZM macromolecules on the hemisphere of an SV by assuming that the multiple AZM macromolecules are randomly distributed but limited to the lower half hemisphere of a vesicle (see Fig. 2). Then simulations were carried out as described above.

### Parameters

An important parameter of the AZM-medicated SV docking and priming model is the number of the AZM macromolecules, their locations on the vesicle surface, and the proximity of the vesicle’s center to the PM. Because the total number of the AZM macromolecules (*n*) can vary, we used *n* ranging from 4 to 10, which is similar to the range obtained from a recent study on 101 docked SVs at active zones of resting frog’s neuromuscular junctions^4^. These numbers are within the range of the number of required SNARE complexes for the fusion of an SV with the PM^34–40^. For the initial proximity of each vesicle’s center to the PM we chose a value of 1.3, which agrees with the distances from centers of the undocked SVs to the PM at active zones of resting frog’s neuromuscular junctions that were normalized with respect to the vesicles’ radius.

### SV shape

The deformed shape of each simulated SV is determined by the distance from the center of the contact area of the SV with the PM to the farthest position of the SV, which is defined as the short diameter of the SV.

### Statistical Analysis

All the statistical analyses were performed with OriginPro (OriginLab, Northampton, MA, USA). Pearson correlation test was used to examine correlations of the contact area with the height of the AZM connection sites and the deformed shape of docked SVs. All the averages were given with their standard deviation.

## Results

### The random fluctuation of the distance from the connection sites of AZM macromolecules on an SV to the PM

We first generated 500 simulated undocked SVs away from the PM. Here we used normalized distances with respect to the radius of an SV because the vesicle size varies broadly depending on the kind of synapses (~30 nm to ~80 nm in diameter)^4, 41–43^, but they can be easily converted to real distances by multiplying the radius of an SV. At resting frog’s neuromuscular junctions, undocked SVs closely located within 40 nm from the PM are rare at active zones because most of them are docked with the PM (~98%); Since the least distance from the membrane of each of the undocked SVs to the PM is ~8 nm, the dimensionless proximity of the center of the vesicles to the PM is ~1.3 using the average diameter of an SV (55 nm)^4^. At hippocampal synapses of mouse brain, the average diameter of an SV is ~45 nm, and it was proposed that SV docking begins when the distance from the SV membrane to the PM is ~6 nm^44^; thus, the distance from the center of such undocked SVs to the PM, which is ~29 nm, can be also converted to 1.3. Then, multiple connection sites from the AZM macromolecules directly linking their SV to the PM are assumed to be randomly located on the hemisphere of each SV facing the PM.

Here we used 4 to 10 AZM macromolecules for each vesicle because various numbers of AZM macromolecules were found to be associated with docked SVs from a recent electron tomography study on frog’s neuromuscular junction^4^. The position of each AZM connection site is assumed to randomly rise or fall from the PM while it is fixed on the vesicle membrane. To simulate the random variation in the height of the connection sites, a Markov Chain Monte Carlo approach was used as described in Methods. After best fitting the vesicle to the changed positions of the AZM connection sites, the positions of the AZM connection sites on the best fit vesicle are set to be their new positions. In this way, the vesicle randomly moves up and down from the PM. We first targeted to obtain the equilibrium distribution of the average height of the AZM connection sites by repeating the random shifting of the AZM connection sites for 80000 times to ensure that the stable distribution of the average height is obtained, and using the average height we calculated the proximity of the SV’s center to the PM and the contact area of the vesicle with the PM.

Figure 3a shows three different distributions of the AZM connection sites randomly located on the hemisphere of an SV facing the PM. Figure 3b,c show that the fluctuation and the distribution of the average height of the AZM connection sites depend on the distribution of the AZM connection sites. In Fig. 3c, the histograms of the average heights for the three SVs have unimodal peaks centered at 0.47 ± 0.037, 0.49 ± 0.038 and 0.45 ± 0.030, respectively. When the random distribution of the AZM connection sites are limited to the upper half hemisphere in Fig. 3d, the average height of the AZM connection sites fluctuates in Fig. 3e, and the distribution of the average height of each of the vesicles is unimodal in Fig. 3f (0.48 ± 0.046, 0.48 ± 0.043, and 0.040 ± 0.032, respectively) similar to Fig 3c. When eight AZM connection sites for each vesicle are randomly distributed on the lower half of the vesicle’s hemisphere as shown in Fig. 3g, the average height of the AZM connection sites fluctuates in Fig. 3h, and the equilibrium probability distribution of the average height is unimodal in Fig. 3i (0.26 ± 0.023, 0.24 ± 0.021, and 0.20 ± 0.017, respectively). These results show that the equilibrium probability distribution of the average height of the AZM connection sites directly depends on the distribution of the AZM connection sites on the vesicle surface. Specifically, the lower the overall distribution of the AZM connection sites is, the smaller the width of the distribution of the average height of the connection sites is as shown in Fig. 3. This is reasonable because each AZM macromolecule is expected to have almost fully stretched structure when its associated SV is at its initial position where it begins docking and the vesicle will preferentially move toward the PM.

### The height of the AZM macromolecules regulates the proximity of their associated SV to the PM and its contact area with the PM

**Figure 4:**
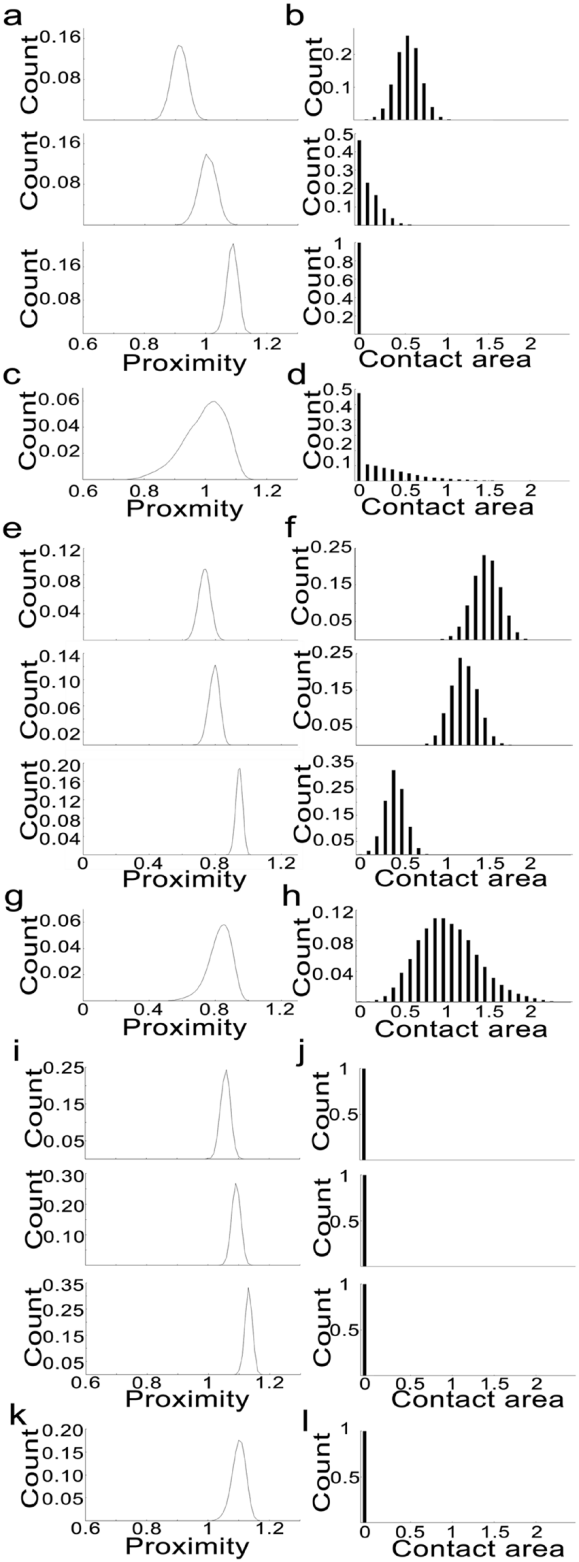
The histograms of the proximity of the center of each of the nine SVs as shown in Fig. 3, the histograms of their contact areas, and the histograms of the proximities and contact areas of 500 simulated SVs. (**a,e,i**) Histograms of the proximity of each of three different SVs having eight AZM connection sites randomly distributed on the surface of an SV’s hemisphere, upper half hemisphere, and lower half hemisphere, respectively as shown in Fig. 3. Note that the distribution of the AZM connection sites on the vesicle surface is related to the distribution of the proximity of the vesicle. (**b,f,j**) Histograms of the contact area of the vesicles. Note that the portion of undocked SVs having 0 contact area and the overall distribution of the contact area are also related to the distribution of the AZM connection sites on the vesicle surface. (**c,g,k**) Histograms of the proximities of 500 simulated SVs having eight AZM connection sites randomly distributed on the surface of each SV’s hemisphere, upper half hemisphere, and lower half hemisphere, respectively as shown in Fig. 3. (**d,h,l**) Histograms of the contact areas of the 500 simulated SVs having eight AZM connection sites randomly distributed on the surface of each SV’s hemisphere, upper half hemisphere, and lower half hemisphere, respectively as shown in Fig. 3.

We use the proximity of an SV to the PM and the contact area of the SV with the PM to quantify the spatial relationship of the SV with the PM. The proximity of an SV to the PM is defined as the distance from the SV’s center to the PM, and the contact area is the area of the PM intersected by the SV. Due to the fluctuating height of the AZM macromolecules (see Fig. 3b,e,h) and their stable location on their connected SV, the proximity and the contact area are expected to vary constantly. The proximity of each of the simulated vesicles to the PM, which is determined by the height of the AZM connection sites (see Methods), is unimodal as shown in Fig. 4a (0.91 ± 0.027, 1.0 ± 0.028, and 1.08 ± 0.019, respectively), and the vesicles’ contact areas show broad variations in Fig. 4b (0.55 ± 0.15, 0.14 ± 0.11, and 0.029 ± 0.016, respectively). The normalized total distribution of the proximity of 500 SVs in Fig. 4c shows that 51% of SVs are docked with the PM. This docking efficiency (51%) is significantly lower than the observed docking efficiency from a recent electron tomography study on frog’s neuromuscular junctions (~98%)^4^. The distribution of the contact area in Fig. 4d shows that the contact area is not normally distributed and that as the contact area increases the probability of having the extent of the contact area decreases monotonously. In contrast, when the random distribution of the AZM connection sites are limited to the upper half hemisphere in Fig. 3d, the docking efficiency of an SV increases greatly to 99.97%, which is comparable to the high docking efficiency of SVs at the active zone from frog’s neuromuscular junctions (~98%)^4^. The proximity of the center of each of the vesicles to the PM is also unimodal as shown in Fig. 4e (0.73 ± 0.038, 0.79 ± 0.034, and 0.94 ± 0.021, respectively), and the vesicles’ contact areas show broad variations as shown in Fig. 4f (1.47 ± 0.17, 1.2 ± 0.17, and 0.37 ± 0.12, respectively). The normalized total distribution of the proximity of the center of all of the 500 SVs predicts that almost all of SVs (99.97%) are docked with the PM as shown in Fig. 4g. The distribution of the contact area in Fig. 4h shows that the distribution of the contact area is unimodal (1.0 ± 0.36). Conversely, when the distribution of the AZM connection sites are limited to the lower half of the hemisphere of an SV, the docking efficiency of the vesicle decreased markedly down to almost 0% indicating that the distribution of the AZM connection sites is an important factor to regulate the efficiency of SV docking. The proximity of each of the SVs to the PM is also unimodal as shown in Fig. 4i (1.05 ± 0.017, 1.08 ± 0.015, and 1.12 ± 0.012, respectively), and it shows that the docking efficiency of an SV is extremely low in Fig. 4j. The total distribution of the proximity of the center of all of the 500 SVs in Fig. 4k,l shows that almost all of SVs (99.98%) are undocked with the PM.

### Correlation of an SV’s contact area with the height of AZM connection sites depends on the distribution of the connection sites on SV

**Figure 5:**
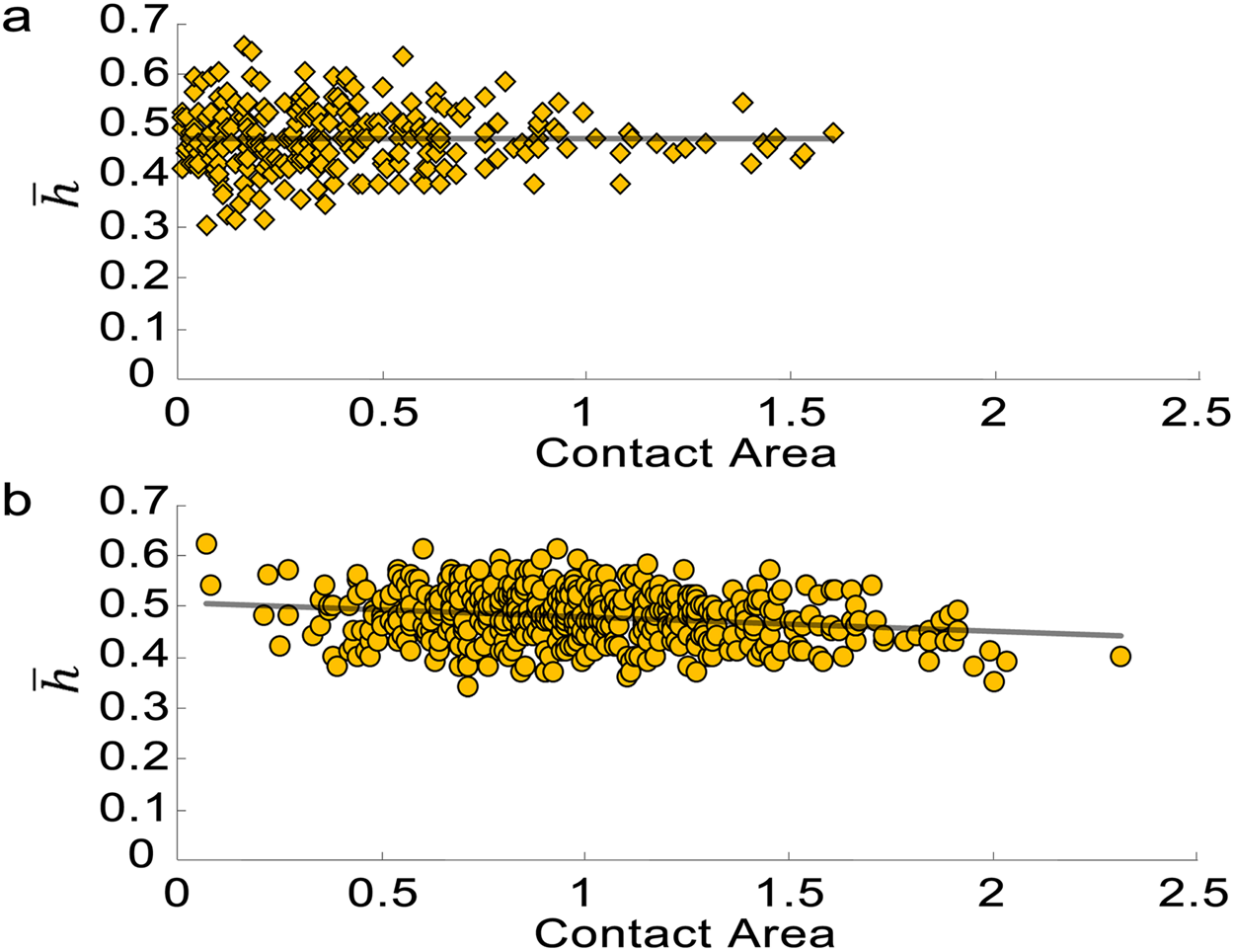
Scatter plot of the average height of the AZM connection sites ($\bar{{\boldsymbol{h}}}$) as a function of the contact area of the vesicle with the PM. (**a**) When the AZM connection sites are randomly distributed on the hemispherical surface of a vesicle facing the PM, the contact area of the vesicle has no significant correlation with the average AZM connection sites based on the results of the 500 simulated SVs (r = 0.0019, p = 0.98). It should be noted that only 266 SVs out of 500 simulated SVs are docked with the PM. (**b**) However, when the AZM connection sites are randomly distributed on the upper half of the hemisphere of an SV, the contact area is correlated with the average AZM connection sites based on the results of the 500 simulated SVs (r = −0.21, p = 3.4 × 10^−6^). Conversely, when the AZM connection sites are randomly distributed on the lower half hemisphere of an SV, based on the results of the 500 simulated SVs there are almost no docked SVs (<0.03%) indicating that the positions of the AZM connection sites on the vesicle surface influence on the portion of the docked SVs (see Fig. 4j).

To gain a deeper understanding of the relationship between the contact area of a docked SV with the PM and the height of connection sites of the AZM macromolecules, we performed correlation analyses. The scatter plots of the contact area vs. the average height of the AZM connection sites are shown in Fig. 5. When eight AZM connection sites are randomly distributed on the hemisphere of each of 500 simulated SVs, the contact area is not correlated with the average height of the AZM connection sites (r = 0.0019; p > 0.05, Pearson correlation test). However, when the connection sites are randomly distributed on the upper half hemisphere of each of 500 simulated SV, the contact area is correlated with the average height of the AZM connection sites (r = −0.21; p < 0.05, Pearson correlation test). We did not execute the correlation analysis for the 500 simulated vesicles having the random distributions of eight AZM connection sites on the lower half of the hemisphere because the probability of the vesicles in contact with the PM is extremely low (<0.03%).

We also examined the correlation of the contact area with the average height of the AZM connection sites as the number of the connection sites varies from four to ten, and we obtained similar results (see Supplementary Table S1). The contact area is not correlated with the average height of the AZM connection sites when the connection sites are randomly distributed on the hemisphere of each of 500 simulated SVs (p > 0.05, Pearson correlation test); however, the contact area is correlated with the average height of the AZM connection sites when the connection sites are randomly distributed on the upper half hemisphere of each of 500 simulated SV (p < 0.05, Pearson correlation test). We did not carry out the correlation analysis for 500 simulated vesicles having the random distributions of eight AZM connection sites on the lower half of the hemisphere because the probability of the vesicles in contact with the PM is extremely low similar to the result when the number of AZM connection sites is eight.

### Correlation of an SV’s contact area with the shape of the SV

**Figure 6:**
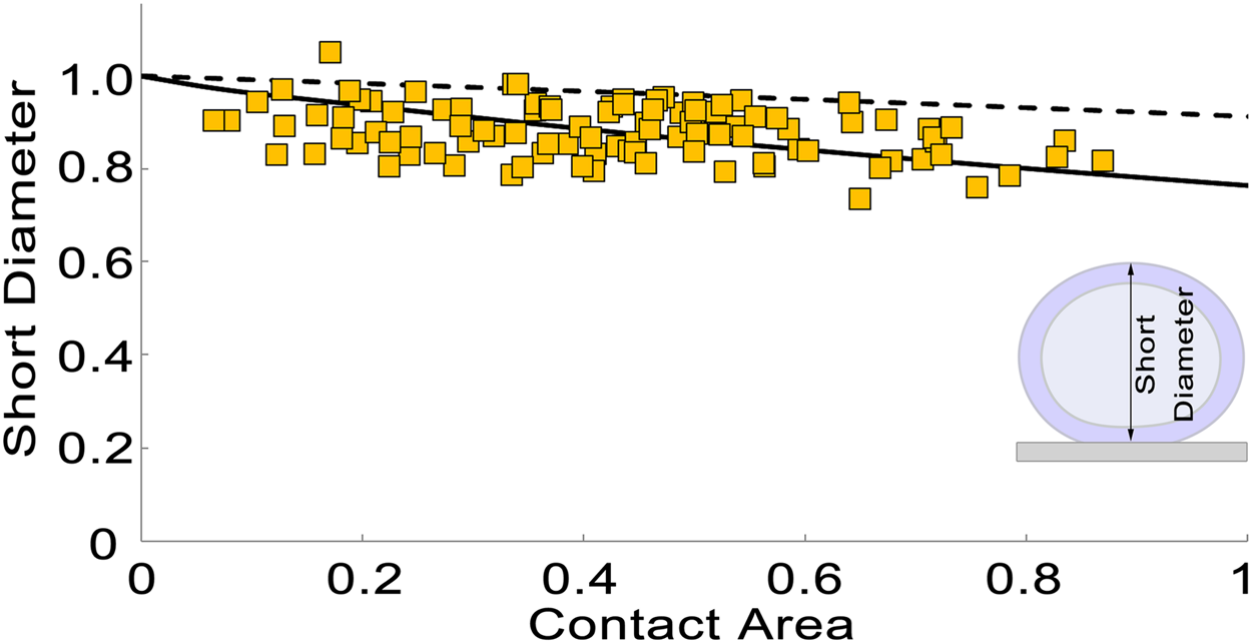
Relationship of an SV’s short diameter with the SV’s contact area at resting active zones of frog’s neuromuscular junctions. Contact areas of 101 docked SVs with the PM at active zones from resting frog’s neuromuscular junctions are plotted against their measured short diameters. The contact area of the docked SVs is correlated with the short diameter of the SVs (Pearson correlation, r = −0.33, and p = 6.8 × 10^–4^). The inset diagram shows the length and orientation of the short diameter for a docked SV. The dotted black line represents an expected relationship of each SV’s contact area with its short diameter from our simulated SVs assuming each SV has a shape of a spherical cap after docking with the PM, and it does not agree with the data. The solid black line is generated from a simple continuum model for docked vesicles assuming the axisymmetric shape and constant surface area of each vesicle and that the vesicle’s elastic energy is equal to the membrane bending energy (see Supplementary Material). The black line shows a significantly improved agreement with the measured relationship of the contact area with the short diameter from the 101 docked SVs^4^.

It is certain that the simulated SV’s contract area with the PM is correlated with the shape of the SV, which can be represented by the distance from the top of the SV to the PM because our model assumes a spherical shape for an undocked SV and a spherical cap for the SV after docking without changing its diameter of the SV’s spherical shape. Numerous theoretical and experimental studies on bilayer lipid vesicles showed that the contact area of a vesicle is closely related to the vesicle’s shape^45–49^. Consistently, assuming that the shape of an SV can be represented by three orthogonal diameters, the ratio of the shortest diameter to the longest diameter of SVs docked with the PM at resting active zones of frog’s neuromuscular junctions was found to be correlated with the SVs’ contact area with the PM^4^. From the 101 docked SVs used for the study^4^, the normalized contact area of an SV is found to be correlated with the short diameter of the SV oriented nearly vertical to the contact site (r = −0.33; p < 0.05, Pearson correlation test) as shown in Fig. 6. However, the expected relationship of the contact area with the short diameter of docked SVs based on our assumption of spherically shaped SVs does not agree with the measured relationship of them in Fig. 6. The difference might be due to the shape change of SVs that occurs after SV docking and the asymmetric distribution of the connection sites of the AZM macromolecules connected to the SVs, which are likely to exert force on the membranes of the SVs asymmetrically. Here a theoretical relationship between the contact area and the short diameter of docked SVs is obtained by a simple continuum model for vesicles consisting of symmetric bilayers that predicts a continuous shape change of a docked SV as its contact area changes^47–50^, and interestingly, it shows a markedly better agreement with the relationship between the contact area and the short diameter of the 101 docked SVs at active zones from frog’s neuromuscular junctions as shown in Fig. 6. Our findings indicate that SVs after docking continue to change in their shape as their contact area with the PM alters.

## Discussion

**Figure 7:**
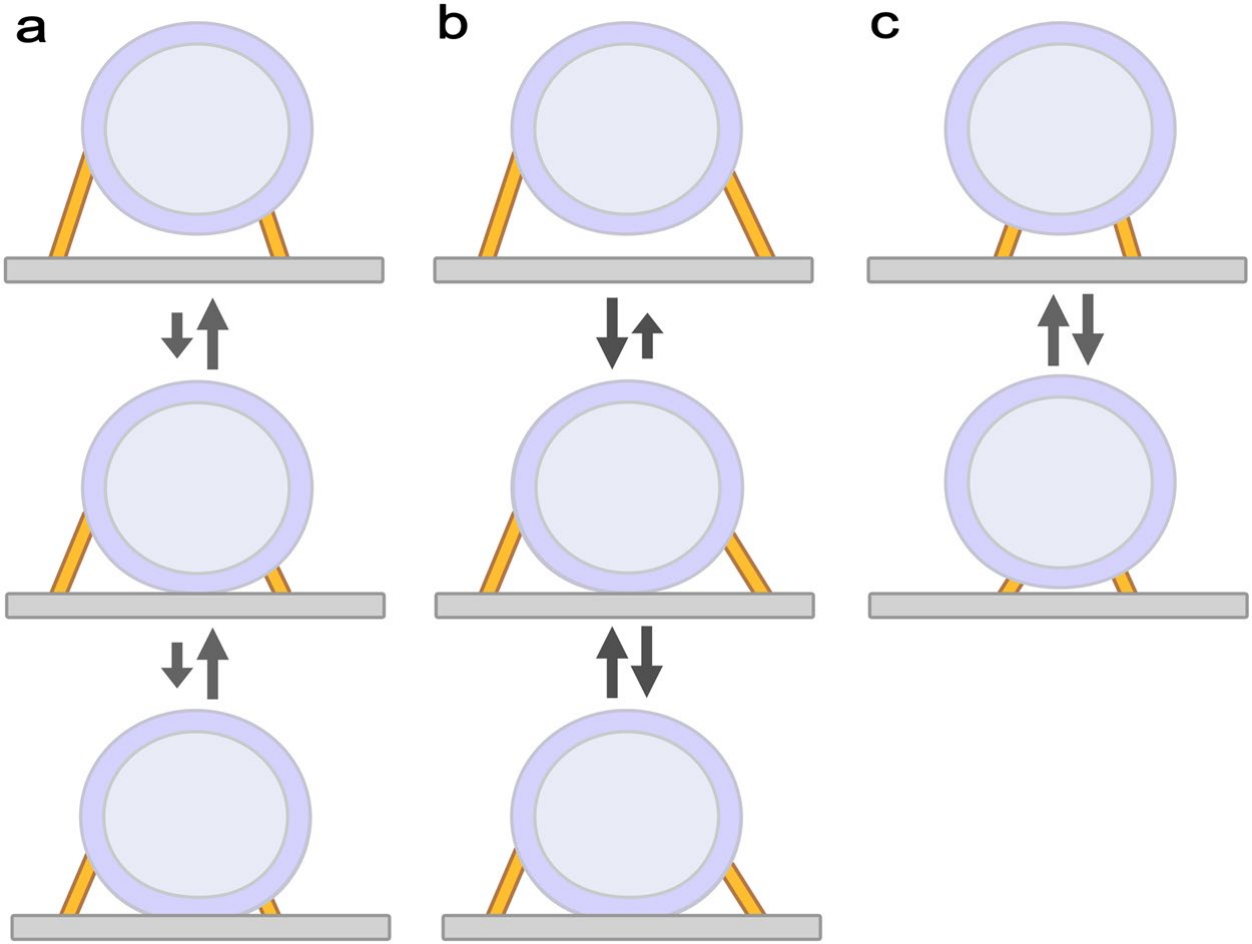
Schematic diagrams of the stochastic AZM mediated SV docking and priming model. Diagrams depict the transition of an SV between undocked and docked states based on the stochastic AZM-mediated SV docking and priming model. Multiple AZM macromolecules dock their associated SV onto the PM and prime it by shortening of the macromolecules, and the SV can be also undocked by lengthening of the macromolecules and repulsive forces between the vesicle membrane and the PM. (**a**) The AZM macromolecules are randomly attached to the hemisphere of the SV facing the PM. Their random distribution combined with their random shortening/lengthening results in moderate docking efficiency (~50%). (**b**) The AZM macromolecules are randomly attached to the upper half of the SV’s hemisphere. Their distribution combined with their random shortening/lengthening results in high docking efficiency (~100%). (**c**) The AZM macromolecules are randomly attached to the lower half of the SV’s hemisphere. Their distribution combined with their random shortening/lengthening results in low docking efficiency (~0%).

We have presented a model of AZM mediated SV docking and priming proposing that the SV docking and priming at resting active zones are regulated by random shortening and lengthening of AZM macromolecules, which directly link SVs to the PM at active zones. Computer simulations based on the model show that the docking efficiency of an SV at the active zone can be greatly altered depending on the distribution of the AZM macromolecules on the SV suggesting that the distribution of the AZM on the SV play an important role on SV release for synaptic transmission. The simulation results also show that the random distribution of the AZM macromolecules on the hemisphere of a SV facing the PM exhibits moderate docking efficiencies (51%). However, when the distribution is limited to the upper half of the vesicle’s hemisphere, the docking efficiencies markedly increase to almost 100% whereas the docking efficiencies fall down to almost 0% with the distribution limited to the lower half of the hemisphere. Thus, the results indicate that the distribution of the AZM macromolecules can significantly influence on the contact area between the vesicle membrane and the PM in addition to the SV docking efficiency as depicted in Fig. 7.

A recent electron tomography study on hippocampal synapses in mouse brain combined with genetic manipulation found that all three members of the SNARE protein family and Munc13 known to be a priming protein^51, 52^ are required for SV docking; it also proposed that membrane-attached vesicles comprise the readily releasable pool^44^, which is in line with the classic SV pool model that SVs in a nerve terminal are postulated to reside in three different pools: a readily releasable pool (docked and primed), a recycling pool, and a reserved pool. SVs in the recycling and reserved pools are recruited for release on moderate and intense stimulation, respectively^53^. In contrast, different electron tomography studies on synapse preparations of rat and mouse brains reported that membrane-attached SVs are rare proposing that SV priming requires short AZM macromolecules or tethers rather than membrane attachment or docking^18, 20^. Thus, the morphological distinction between SV docking and priming is still unclear and might be synapse-specific. However, partially complexed SNARE proteins, which are probably equivalent to short AZM macromolecules, are widely accepted to be involved in SV docking and priming^9^. Consistently, our model shows that shortening of multiple AZM macromolecules can direct SV docking and priming, and it predicts that the distinction between SV docking and priming depends on the distribution of the connection sites of the AZM macromolecules. If connection sites of the AZM macromolecules on the SV membrane are distant from the PM as shown in Fig. 7a,b, the force generated by their shortening is expected to effectively bring the SV membrane and the PM close or in contact. Consistently, a recent electron tomography study on frog’s neuromuscular junctions reported that the combined membrane thicknesses of docked SVs and the PM at their contact site are the same with the sum of their membrane thicknesses away from their contact site between the vesicle membrane and the PM at resting active zones indicating that docked SVs are in contact with the PM without any notable gap or hemifusion^4^, which is a widely accepted fusion intermediate^54^. Similarly, an electron tomography study on hippocampal synapses in mouse brain reported that SVs are docked with the PM without any indication of hemifusion^24^; other electron tomography studies on Caenorhabditis elegans and mouse neuromuscular junctions also reported docked SVs with the PM proposing that only docked SVs constitute the readily releasable pool^55, 56^. In contrast, a few electron tomography studies on synapses in rat brain reported that a significant portion of SVs at active zones are hemifused with the PM^14, 57^. Although the presence of hemifused SVs at resting active zones might depend on kinds of synapses, all of these studies are consistent with the prediction from our model that the contact site between the SV membrane and the PM is devoid of key proteins for SV fusion such as SNARE proteins and their regulatory proteins. Conversely, if the connection sites are relatively closely located from the PM as depicted in Fig. 7c, the AZM macromolecules surround a small region of the SV membrane and will have higher chances to intervene between the vesicle membrane and the PM lowering the docking efficiency. It is recently reported that SVs at active zones from synapse preparations in mouse brain are not in direct contact with the PM proposing that the SVs linked to the PM via several short AZM macromolecules (<~5 nm) are primed^18^. The force generated by shortening of the AZM macromolecules will contribute to changing the curvatures of the SV membrane and the PM surrounded by the macromolecules promoting fusion pore formation between the SV membrane and the PM. Those short AZM macromolecules intervening between the SV membrane and the PM might interact with each other and form a radial SNARE super-complex that has long been envisioned by modeling studies^58–60^.

Several electron tomography studies identified organizations of AZM macromolecules at active zones^4, 12, 14, 16, 17^. In central synapses from rat brain, the polyhedral cage-like arrangement in the AZM has been suggested^14^, and in peripheral synapses, such as mouse and frog’s neuromuscular junctions, the AZM macromolecules at active zones were found to possess well-organized arrangements^12, 16, 17^; furthermore, several classes of the AZM were discovered to display relatively confined distributions on the vesicle membrane^4, 15, 17^. Because the measured average heights of connection sites of such classes of the AZM on 101 docked SVs at resting frog’s neuromuscular junctions are more than ~10 nm, it is reasonable to expect that the AZM connection sites are most likely to be located away from the docking site or contact site of their SV so that the SVs are predicted to have high docking efficiencies based on our model, which agrees well with the measured high SV docking efficiency (~98%)^4^. However, a few other electron tomography studies on central synapses have not reported such organizations at active zones^20, 24^. In the case, our model predicts that SVs at active zones in the synapses have relatively moderate docking efficiencies. Consistently, a study on central synapses from mouse brain showed that a ratio of docked SVs to SVs within 40 nm from the PM at active zones is ~55%^24^, which is comparable to the moderate docking efficiency for the case of random distribution of the AZM on the vesicle membrane predicted by our model.

Biological membranes possess peculiar elastic properties such as bending rigidity, and the elastic properties of membranes are basic parameters that control deformation of the membranes. Significant studies have been focused on theoretical prediction of membrane shape based on the elastic properties and precise evaluation of such elastic properties of membranes^45, 47, 48, 50, 61–66^. We used a simple continuum model of a docked vesicle to have a better understanding of the membrane deformation of docked SVs at active zones (see Supplementary Material), and we discovered that the model showed a better agreement with the measured deformed shapes for docked SVs having various contact areas than a model assuming a shape of spherical cap for the SVs as shown in Fig. 6 (see also Fig. S1 in the Supplementary Material). The finding suggests that docked SVs weakly adhere to the PM because a bound vesicle with constant volume is known to attain the shape of a spherical cap in the limit of strong adhesion^62^ and also suggests that the elastic properties of SVs play an important role on the shape deformation of SVs after docking. When we assume the bending rigidity of the vesicle membrane is 50 k_B_T^67^ (where k_B_ is the Boltzman’s constant and T is temperature), the average adhesion energy for a docked SV having 0.44 as the normalized contact area (345 nm^2^ for an SV having a radius of 28 nm) can be estimated to be 35 k_B_T; for a docked SV having 1.0 (784 nm^2^ for an SV having a radius of 28 nm) is 100 k_B_T (see Supplementary Material). Because a recent study reported that the formation of a partial SNARE complex leads to a net energy release of ~26 k_B_T^68^, the shortening of multiple AZM macromolecules presumably by partial SNARE complex assembly is expected to provide sufficient energy for a docked SV to have the reported range of their contact areas with the PM from ~50 nm^2^ to ~650 nm^2^ at active zones of frog’s neuromuscular junctions, and the energy generated by shortening of the macromolecules may also contribute to SV priming by promoting the membrane destabilization within the contact site between the SV membrane and the PM^4^.

In this study we proposed a stochastic AZM-mediated SV docking and priming model by extending the AZM-mediated variable force hypothesis for a docked SV’s priming recently proposed^4^. Simulation results based on the model showed that the arrangement of connection sites of AZM macromolecules directly linking their SV to the PM has important implications for spatial relationships of the SV with the PM such as the proximity of the SV to the PM and the contact area between the SV and the PM as depicted in Fig. 7. The model offers experimentally testable morphological features in the spatial relationships of SVs with the PM at active zones in any synapses that may contribute to reconciling seemingly contradictory observations regarding SV docking and priming, which are critical for SV exocytosis^4, 12, 18, 20^. Therefore, testing the model by further examination of the spatial relationship of SVs with the PM at active zones in various synapses will contribute to developing common mechanisms of SV docking and priming that can be generally applicable to any synapses.

## Electronic supplementary material

Supplementary information
