# Supplementary material for: A stochastic model of active zone material mediated synaptic vesicle docking and priming at resting active zones
Source: Sci Rep. 2017 Mar 21;7:278. doi: 10.1038/s41598-017-00360-z (PMC5428245; doi:10.1038/s41598-017-00360-z)
Supplement: Supplementary file 1 — Supplementary information [file 41598_2017_360_MOESM1_ESM.pdf]

A stochastic model of active zone material mediated synaptic vesicle docking and priming at resting active zones

Jae Hoon Jung[†‡*] and Sebatian Doniach[†]

[†]Department of Physics, Stanford University School of Humanities and Sciences, Stanford, CA 94305

[‡]Department of Biology, Texas A&M University, College Station, TX 77845

[*]jjung@bio.tamu.edu

SUPPORTING MATERIAL

Because most of the methods about sample preparation, electron tomography, and electron tomographic data analyses related to the presented data in Fig. 6 are described in [1], we briefly describes the methods here.

Sample Preparation

Resting frog's neuromuscular junctions were prepared for electron tomography according to the methods described in [1-3]. Briefly, cutaneous pectoris muscles of adult Rana pipiens (about 5cm nose-rump length) were fixed with 0.8%-1% glutaraldehyde (Ted Pella, Inc., Redding, CA) in Millonig's phosphate buffer, refixed and stained with 1% osmium tetroxide in phosphate buffer, stained with saturated aqueous uranyl acetate, dehydrated in ethanol and propylene oxide, and embedded in Epon 812 or Eponate 12 (Ted Pella, Inc., Redding, CA). Thin sections (~50 nm to ~150 nm) were mounted on Formvar or polyetherimide coated 1 x 2 mm single slot grids stained with saturated uranyl acetate, followed by lead citrate. For use in the alignment of tilt images, stained and mounted sections were floated on a drop of ~ 0.01% 5 nm or 10 nm unconjugated gold colloid in H_2O (Sigma Chemical Co., St Louis, MO) for 1-2 minutes and air dried.

Electron Tomography

We used 32 datasets of tilt images collected using one of two electron microscopes designed for automatic data acquisition: 1) FEI TF30 Polara electron microscope (FEI Company Hillsboro, OR) equipped with a 2048x2048 Tietz TemCam-F224HD CCD (Tietz Video and Imaging Processing Systems GmbH, Gauting, Germany); and 2) FEI Tecnai G2 F20 electron microscope (FEI Company Hillsboro, OR) equipped with a 2048x2048 Gatan CCD (Gatan, Inc., Pleasanton, CA). The stage on each microscope was cooled to liquid N_2 temperature to reduce beam damage to the specimen. Datasets consisted of images taken at 1° or 2° tilt intervals to about ±60° to ±70° along a single tilt axis or two orthogonal tilt axes. The tilt-images were aligned automatically using 5 or 10 nm gold colloid (British Biocell International, Cardiff, U.K.) deposited on one or both sides of the sections as fiducial markers before data collection. A reconstructed volume from each set of aligned tilt-images was generated using a weighted back-projection method. Both the alignment and reconstruction algorithms are in the unified software package for electron tomography, EM3D (em3d.org) [4]. Structures were segmented from the reconstruction volumes using EM3D to define individual volumes-of-interest (VOIs) and they were rendered to generate 3D surface models, as described in [4].

Electron Tomographic Data Analysis

SV size and its deformed shape. The 3D shape of each SV surface model was determined by measuring three orthogonal diameters along the three principle axes of the SV shape [1]. To determine the size of each docked SV in our datasets, three orthogonal dimeters denoted by d_l, d_i, and d_s were used to calculate a geometric average according to the following equation:

$$\text{SV size } = (d_l d_s d_i)^{1/3} \quad \text{(S1).}$$

A diameter, which is the most vertical to the PM among the three diameters was selected as an indicator of SV's deformed shape toward the PM, as presented in the inset of Fig. 6. The

diameter is normalized by dividing it by the long diameter, which is the greatest diameter among the three orthogonal diameters.

Extent of the contact area of an SV with the PM. The vertices at the interface of the vesicle membrane and PM at their contact site were projected onto the best-fit plane along an eigenvector containing the least eigenvalue, which was calculated using the covariance matrix of the vertices' coordinates. The best-fit plane was pixilated, and each pixel was standardized to have the area of one face of a voxel from the reconstruction to maintain scale. The contact area was calculated by counting the number of pixels that contained the projected vertices and converting it to area according to scale. The contact area was normalized by dividing it by the total surface area using the vesicle size.

Correlation Analysis of an SV's contact area with the average height of the AZM connection sites.

For each of 500 simulated SVs, its contact area and average height were randomly selected, and using the array of the contact areas and average heights of the 500 SVs, the Pearson correlation coefficient and p-values were obtained. This process was repeated for 10000 times, and the average p-values and the ratio of the number of the cases having no significant correlation ($p \geq 0.05$) to the total number of repetition or 10,000 were obtained as shown in Table S1. Table S1 shows that for all different number of connection sites ranging from four to ten, when the AZM connection sites are randomly distributed on the hemisphere of each of 500 simulated SVs, the contact area is not correlated with the average height of the AZM connection sites (each $p>0.05$). However, when the connection sites are randomly distributed on the upper half of the hemisphere of each of 500 simulated SV, the contact area is correlated with the average height of the AZM connection sites (each $p<0.05$). We did not execute the correlation analysis for the 500 simulated vesicles having the random distributions of four to eight AZM connection sites on the lower half of the hemisphere because the probability of the vesicles in contact with the PM is extremely low (<2%).

Table S1. Correlation analyses of the contact area with the average height the AZM connection sites having various number of connection sites ranging from 4 to 10.

	Random distribution on the hemisphere of an SV		Random distribution on the upper half of the hemisphere of an SV	
Number of the AZM connection sites on an SV	Average p-value	The number of $p \geq 0.05$ over the total number of iteration	Average p-value	The number of $p \geq 0.05$ over the total number of iteration
4	0.6671	0.9993	8.403×10^{-8}	0
5	0.3696	0.9522	1.394×10^{-5}	0
6	0.09621	0.5197	4.053×10^{-10}	0

7	0.5854	0.995	3.500×10^{-4}	0.0008
8	0.4652	0.9783	1.925×10^{-4}	0.0002
9	0.6421	0.9979	1.414×10^{-5}	0
10	0.3056	0.9200	2.351×10^{-6}	0

Note that the total number of iteration is 10000, and each p-value was obtained from Pearson correlation test.

A model of an axisymmetric shape for a docked vesicle with the PM

We used a simple continuum model of a docked vesicle assuming that the vesicle is axisymmetric in shape along the *z*-axis and its surface area is constant [5,6]. For simplicity, we also assumed that the elastic energy stored in a vesicle is the same with the membrane bending energy (cH^2 where c is the bending rigidity and H is the mean curvature of the vesicle membrane) and that the osmotic pressure across the vesicle membrane can be ignored. Our equations come from the membrane model derived in [7]. Here we briefly outline the key features of the model. To obtain the deformed shape of the non-contacting portion of the docked vesicle membrane, a flat circular membrane with radius $2L$ is used as the reference configuration for the non-contacting vesicle membrane ($L \leq R$). The derivation of the governing equations can be found in [7]. We use a cylindrical coordinate system (r, θ, z) to represent a shape of the deformed vesicle membrane where θ is the azimuthal angle about the z-axis. Since we consider axisymmetric deformation, the deformed membrane can be described by two coordinates (s,θ) where s is the arc length of the cross-sectional curve. S, which is a function of s, is the corresponding arc length in the reference configuration, which is not deformed. The angle made by the tangent of the deformed cross-section curve with the z-direction is denoted by φ.

By introducing the normalized S $\left(\bar{S} = \frac{S}{L}\right)$, the normalized equilibrium equations, which were derived in [7] are:

$$\frac{dq}{d\bar{S}} = -q\frac{\bar{S}}{\bar{r}^2}\sin\varphi - 2h\frac{\bar{S}}{\bar{r}}\left[\bar{d} + h^2 + \frac{\cos\varphi}{\bar{r}}\left(2h + \frac{\cos\varphi}{\bar{r}}\right)\right] \quad \text{(S2)}$$

$$\frac{dh}{d\bar{S}} = q\frac{\bar{S}}{\bar{r}} \quad \text{(S3)}$$

$$\frac{d\varphi}{d\bar{S}} = \left(2h + \frac{\cos\varphi}{\bar{r}}\right)\frac{\bar{S}}{\bar{r}} \quad \text{(S4)}$$

$$\frac{d\bar{r}}{d\bar{S}} = \frac{\bar{S}}{\bar{r}}\sin\varphi \quad \text{(S5)}$$

$$\frac{d\bar{z}}{d\bar{S}} = \frac{\bar{S}}{\bar{r}}\cos\varphi \quad \text{(S6)}$$

where q is the normalized transverse shear force and h the normalized mean curvature ($q = \frac{QL^2}{c}$ and $h = HL$). $\bar{r}$ and $\bar{z}$ are the normalized coordinates of the non-contacting vesicle membrane. Thus, there are five coupled differential equations for unknown $h, q, \bar{r}, \varphi,$ and $\bar{z}$. Because of symmetry in the assumed shape of the docked vesicle, the shear force (q) is 0 when $\bar{S}$=0. The slope is zero at both ends of the cross-sectional curve where $\bar{S}$=0 and 2. Thus, $\varphi(\bar{S} = 0) = \frac{\pi}{2}$ and $\varphi(\bar{S} = 2) = \frac{3\pi}{2}$. Also, $r(\bar{S} = 0) = 0$ and $\bar{z}(\bar{S} = 2) = 0$.

In equilibrium, the adhesion energy, W_{ad} is assumed to balance the elastic bending energy at $\bar{S}$=2. In other words, a dimensionless adhesion energy is

$$h(\bar{S} = 2) = \sqrt{\frac{W_{\mathrm{ad}}L^2}{c}} = \frac{L}{R}\sqrt{\alpha} \quad \text{(S7)}$$

where $= \frac{W_{ad}R^2}{c}$.

Using the five boundary conditions and $h(\bar{S} = 2) = \frac{L}{R}\sqrt{\alpha}$, the five differential equations was solved using the boundary value problem solver, bvp4c in Matlab. Figure S1 shows the change in the deformed vesicle membrane for various values of α.

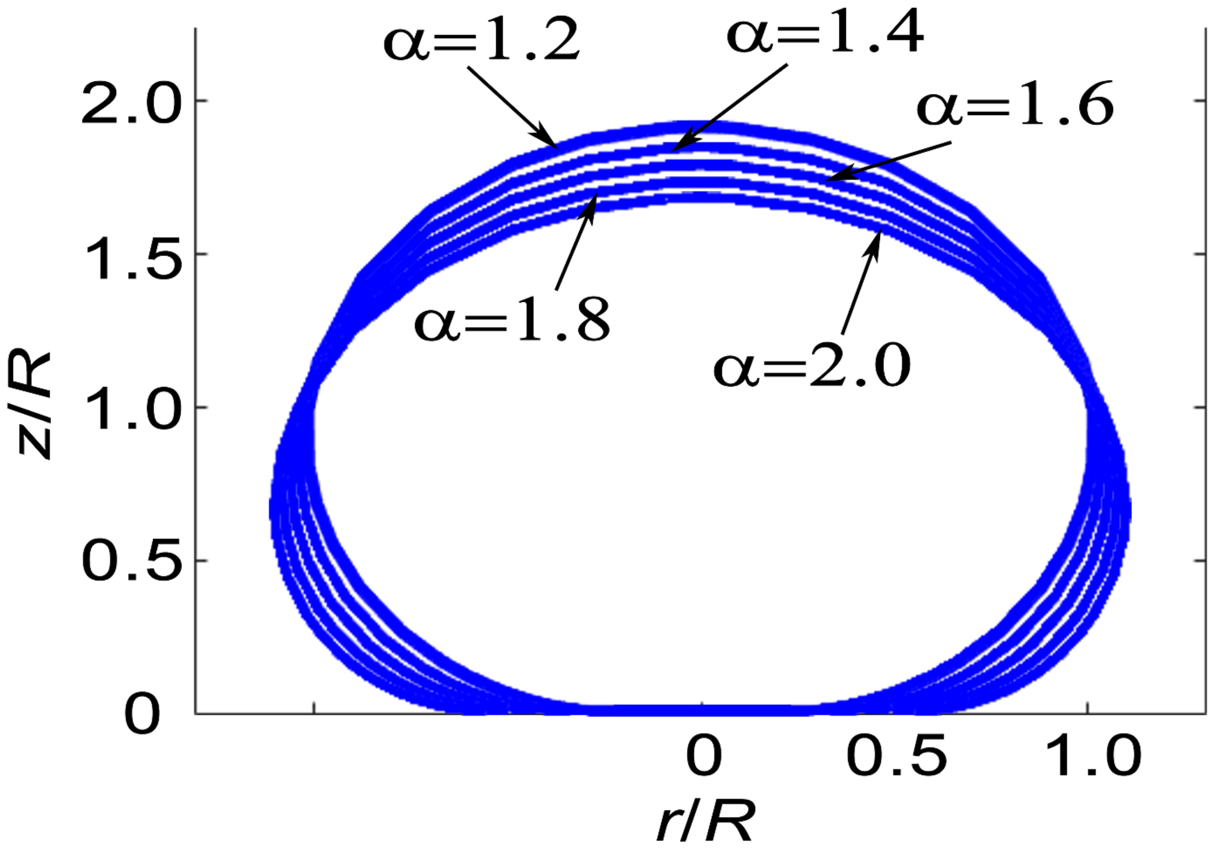

Figure S1. Cross-section shape of a docked SV for α=1.2, 1.4, 1.6, 1.8, and 2.0. The cross-section shape of a docked SV varies as α changes. Note that the contact area is dependent on the value of α (contact area = 0.17, 0.39, 0.44, 0.81, and 1.0 for α=1.2, 1.4, 1.6, 1.8, and 2.0, respectively).

Supporting References

1 Jung, J. H., Szule, J. A., Marshall, R. M. & McMahan, U. J. Variable priming of a docked synaptic vesicle. *Proc Natl Acad Sci U S A* **113**, E1098-1107, doi:10.1073/pnas.1523054113 (2016).

2 Harlow, M. L., Ress, D., Stoschek, A., Marshall, R. M. & McMahan, U. J. The architecture of active zone material at the frog's neuromuscular junction. *Nature* **409**, 479-484, doi:10.1038/35054000 (2001).

3 Szule, J. A. *et al.* Regulation of synaptic vesicle docking by different classes of macromolecules in active zone material. *PLoS One* **7**, e33333, doi:10.1371/journal.pone.0033333 (2012).

4 Ress, D. B., Harlow, M. L., Marshall, R. M. & McMahan, U. J. Methods for generating high-resolution structural models from electron microscope tomography data. *Structure* **12**, 1763-1774, doi:10.1016/j.str.2004.07.022 (2004).

5 Seifert, U. & Lipowsky, R. Adhesion of vesicles. *Phys Rev A* **42**, 4768-4771 (1990).

6 Lipowsky, R. & Sackmann, E. *Structure and dynamics of membranes*. (Elsevier Science, 1995).

7 Long, R., Hui, C. Y., Jagota, A. & Bykhovskaia, M. Adhesion energy can regulate vesicle fusion and stabilize partially fused states. *J R Soc Interface* **9**, 1555-1567, doi:10.1098/rsif.2011.0827 (2012).
